# Preventing Ischemic Cerebrovascular Events in High-Risk Patients With Non-disabling Ischemic Cerebrovascular Events Using Remote Ischemic Conditioning: A Single-Arm Study

**DOI:** 10.3389/fneur.2021.748916

**Published:** 2021-12-16

**Authors:** Shimeng Liu, Zongen Gao, Ran Meng, Haiqing Song, Tianping Tang, Ya Zhao, Rong Chen, Yanzhen Sheng, Qianqian Fan, Fang Jiang, Qian Zhang, Jianping Ding, Xiaoqin Huang, Qingfeng Ma, Kai Dong, Sufang Xue, Zhipeng Yu, Jiangang Duan, Changbiao Chu, Xiaohui Chen, Xingquan Huang, Sijie Li, Bruce Ovbiagele, Wenle Zhao, Xunming Ji, Wuwei Feng

**Affiliations:** ^1^Department of Neurology, Xuanwu Hospital, Capital Medical University, Beijing, China; ^2^Department of Neurology, Beijing Tiantan Hospital, Capital Medical University, Beijing, China; ^3^Department of Neurology, Shengli Oilfield Center Hospital, Dongying, China; ^4^Department of Neurology, Taoyuan People's Hospital, Changde, China; ^5^Department of Neurology, First Affiliated Hospital of Hainan Medical University, Haikou, China; ^6^Departmeng of Neurology, University of California, San Francisco, San Francisco, CA, United States; ^7^Department of Public Health Sciences, Medical University of South Carolina, Charleston, SC, United States; ^8^Beijing Key Laboratory of Hypoxia Conditioning Translational Medicine, Beijing, China; ^9^Center of Stroke, Beijing Institute for Brain Disorders, Beijing, China; ^10^Cerebrovascular Diseases Research Institute, Xuanwu Hospital of Capital Medical University, Beijing, China; ^11^China-America Institute of Neuroscience, Xuanwu Hospital, Capital Medical University, Beijing, China; ^12^Institute of Hypoxia Medicine, Xuanwu Hospital, Capital Medical University, Beijing, China; ^13^Laboratory of Brain Disorders, Beijing Institute of Brain Disorders, Ministry of Science and Technology, Collaborative Innovation Center for Brain Disorders, Capital Medical University, Beijing, China; ^14^Beijing University of Aeronautics & Astronautics-China Capital Medical University (BUAA-CCMU) Advanced Innovation Center for Big Data-Based Precision Medicine, Beijing, China; ^15^Department of Neurology, Duke University School of Medicine, Durham, NC, United States

**Keywords:** remote ischemic conditioning, stroke, transient ischemic attack, secondary prevention, acute minor ischemic stroke, transient ischemic attack

## Abstract

**Background:** Secondary stroke prevention after a high-risk, non-disabling ischemic cerebrovascular event needs to be enhanced. The study was conducted to investigate whether remote ischemic conditioning (RIC) is effective in preventing recurrent ischemic events within 3 months.

**Methods:** This was a four-center, single-arm, open-label Phase IIa futility trial (PICNIC-One Study). Adult patients (≥18 years of age) who had an acute minor ischemic stroke (AMIS) with a National Institutes of Health Stroke Scale score ≤ 3 or a transient ischemic attack (TIA) with moderate-to-high risk of stroke recurrence (ABCD score ≥ 4) within 14 days of symptom onset were recruited. Patients received RIC as adjunctive therapy to routine secondary stroke prevention regimen. RIC consisted of five cycles of 5-min inflation (200 mmHg) and 5-min deflation of cuffs (45 min) on bilateral upper limbs twice a day for 90 days.

**Results:** A total of 285 patients met the study criteria, of which 167 provided signed informed consent and were enrolled. Data from 162 were analyzed with five subjects excluded. Recurrent AIS/TIA occurred in 6/162 (3.7%) patients within 3 months, with no occurrence of hemorrhagic stroke. The top three adverse events were upper limb pain (44/162, 27.2%), petechia (26/162, 16.0%), and heart palpitation (5/162, 3.1%). About 68 (42.0%) subjects completed ≥ 50% of 45-min RIC sessions.

**Conclusions:** RIC is a safe add-on procedure and it has a potential benefit in reducing recurrent cerebrovascular events in patients with high-risk, non-disabling ischemic cerebrovascular events as the risk of stroke/TIA events is lower than expected; however, its compliance needs to be improved. Our study provides critical preliminary data to plan a large sample size, randomized controlled clinical study to systematically investigate the safety and efficacy of RIC in this population.

## Introduction

Stroke is the most common cause of mortality and disability in China (1). High-risk non-disabling ischemic cerebrovascular events consist of acute minor ischemic stroke (AMIS) and moderate-to-high risk transient ischemic attack (TIA). The most commonly used definition of AMIS is a National Institutes of Health Stroke Scale (NIHSS) score <4 at the time of the event (2).

In particular, a large-scale clinical trial among Chinese patients with AMIS/TIA (CHANCE study) has indicated that dual antiplatelet therapy (clopidogrel and aspirin) reduced the risk of recurrent stroke, as compared to single antiplatelet therapy (3). Subsequently, the latest American and Chinese guidelines for the secondary prevention of stroke has also recommended the combination of aspirin and clopidogrel within 24 h of a high-risk non-disabling ischemic cerebrovascular event, which is continued for 21 days (4, 5). However, according to the CHANCE study results, 9.4% of patients would still have another ischemic stroke/TIA within the next 3 months, despite receiving the recommended dual-antiplatelet therapy (3). Moreover, only fewer than two-thirds of stroke patients were reported to arrive at hospitals within 24 h to receive dual-antiplatelet therapy in China (6). Furthermore, there have been no published data demonstrating whether guidelines have changed real-world clinical practice in China up to our knowledge.

Remote ischemic conditioning (RIC) involves repetitive and brief cuff inflation around the limb to the pressures level above systolic blood pressure and subsequent deflation to induce repetitive ischemia and reperfusion, which has been demonstrated to protect distant organs, such as the heart, kidney, or brain (7–9). Our prior small sample-size, clinical studies have indicated that long-term, regular (twice a day for at least 90 consecutive days), bilateral upper-limb RIC was effective in reducing stroke recurrence in patients with symptomatic intracranial artery stenosis (10, 11). One recent clinical trial in China found repeated remote ischemic post-conditioning during hospitalization combined with intravenous thrombolysis significantly facilitate functional recovery at day 90 in patients with acute ischemic stroke (12). Similarly, another study in UK showed that with 4 cycles of RIC within 24 h of onset of stroke symptoms can lead to a significant decrease in day 90 NIHSS score (13).

Therefore, we hypothesized that adjunctive, twice-daily RIC for 3 months can further reduce cerebrovascular events in patients with AMIS/TIA. To that end, we performed a single-arm, open-label, multi-center, Phase IIa futility study.

## Materials and Methods

A team of researchers from Xuanwu Hospital of the Capital Medical University (China) and the Medical University of South Carolina (USA) designed this study. A detailed rationale and study design of the Preventing Ischemic Cerebrovascular events in patients with acute Non-disabling Ischemic Cerebrovascular events using RIC (PICINIC-One) study was published previously (14). This study was conducted at four sites in China: Xuanwu Hospital of Capital Medical University, Shengli Oilfield Center Hospital, the First Affiliated Hospital of Hainan Medical University, and Taoyuan People's Hospital.

The study was approved by the Institutional Review Board (IRB) of each study site: Xuanwu Hospital Capital, Shengli Oilfield Center Hospital, Taoyuan People's Hospital, and the First Affiliated Hospital of Hainan Medical. Informed consent was obtained from all included participants prior to the study. The study was registered on clinicaltrial.gov (NCT03004820). All procedures in this study were performed in accordance with the ethical standards of the institutional and with the 1964 Declaration of Helsinki and its later amendments or comparable ethical standards.

### Study Population

We recruited adult (≥18 years) patients of either sex, who had an AMIS with NIHSS score ≤ 3 or a TIA with moderate-to-high risk of stroke recurrence (ABCD^2^ Score ≥4) within 14 days of symptom onset.

The inclusion criteria were as follows: (1) ≥18 years of any sex or ethnicity; (2) diagnosis of non-cardiogenic AMIS/TIA within 14 days of stroke symptom onset, wherein AMIS was defined as ischemic stroke with an NIHSS score ≤ 3 at the time of enrollment, and TIA was defined as a transient episode of neurological dysfunction without acute infarction with moderate-to-high risk of stroke recurrence (defined as an ABCD score ≥ 4 at the time of enrollment); (3) stable vital signs, with normal cardiac (Class I-II in New York Heart Association Functional Classification), hepatic (normal ranges in blood liver function tests) and renal functions (normal ranges in blood renal function tests); (4) ability consent by themselves or by a legally authorized representative; and (5) agreement to conduct regular RIC by themselves or others.

On the other hand, subjects who met any of the following exclusion criteria were excluded from the study: (1) diagnosis of brain hemorrhage or other pathologies, such as vascular malformation, tumor, abscess, or other non-vascular diseases, based on brain computed tomography (CT) or magnetic resonance imaging (MRI); (2) modified Rankin Scale (mRS) score >2 before the index event; (3) administration of intravenous thrombolytic therapy (Alteplase or Urokinase) or endovascular treatment for the index event; (4) contraindication to aspirin or clopidogrel (e.g., known allergy, severe asthma, heart failure); (5) indication for anticoagulation therapy (cardiac source of embolus); (6) hemorrhagic tendency of any reason (including but not limited to hemostatic disorder, platelet count <100 × 10^9^/L, history of hepatic dysfunction, among others); (7) any hemorrhagic transformation on brain scans (MRI or CT); (8) gastrointestinal bleed or major surgery within 3 months before the index event; (9) stroke or TIA due to medical procedure or other iatrogenic cause; (10) any upper extremity soft tissue disease, vascular injury, or peripheral blood vessel disease, which is contraindicated for RIC; (11) hypertension with a systolic blood pressure ≥ 200 mmHg despite medical treatment at the time of enrolment; (12) planned revascularization (any angioplasty or vascular surgery) within the following 3 months; (13) scheduled surgery or intervention within the following 3 months that may affect the study procedure; (14) life expectancy ≤ 6 months; (15) pregnant at the time of the study; and (16) ongoing investigational drug or device use by other studies at the time of the study.

### Procedures

Eligible subjects were identified from the inpatient service or stroke emergency center of each study site, where a well-trained research physician confirmed the diagnosis of AMIS (NIHSS score ≤ 3) or TIA (ABCD^2^ score ≥ 4). If the patient met the criteria and provided written informed consent by themselves or legal proxies, they were instructed how to perform RIC using an electric auto-control device (patent number ZL200820123637.X, China), which can be performed by the patient or family members. RIC consisted of five cycles of 5-min inflation at 200 mmHg and 5-min deflation of cuffs on bilateral upper limbs twice a day (45 min) for 90 consecutive days. Antiplatelet strategies based on the physician's best judgment were as follows: aspirin alone (100–300 mg daily), clopidogrel alone (75 mg daily), or a combination of aspirin and clopidogrel. Study visits were conducted on the day of enrollment (day 1) at the inpatient wards or emergency rooms, and the patients were followed up on day 30 ±7 and 90 ±14 in the outpatient department.

### Outcomes

In this study, the primary efficacy outcome was the number (percentage) of patients who had a recurrent ischemic stroke or TIA within 90 days after the index event. The secondary efficacy outcome measures included: (1) number (percentage) of patients who had a second ischemic stroke or TIA within 1 month after the index event; (2) number (percentage) of patients with a new cerebrovascular and coronary artery event within 1 and 3 months, including hemorrhagic stroke, myocardial infarction, and deaths from cardiovascular causes, from all causes, and from the index event; (3) NIHSS score change (continuous) from the baseline to 1 and 3 months; (4) mRS score (continuous), dichotomized by percentage with score ≤ 1 vs. ≥2, at 1 and 3 months; (5) Barthel index score (continuous), dichotomized by percentage with score ≥95 vs. <95, at 1 and 3 months; and (6) hand grip strength change (continuous) from baseline to 1 and 3 months on the affected side in patients with upper-limb motor deficit who did not have a recurrent vascular event.

Additionally, compliance was assessed with simultaneous records, which were delivered by the electric auto-control device through 4G signals, and the feasibility outcomes were defined as the number (percentage) of patients who completed ≥50% or <50% of the long-term, regular, 45-min RIC.

Furthermore, safety outcomes were defined as the risks of expected device-related local or systemic adverse events, including the number (percentage) of patients with upper limb pain assessed by the visual analog scale; redness, swelling, or skin petechiae; palpitation; and dizziness. Any new condition (symptoms, injuries, significant abnormal laboratory values) that was not present at the beginning of the study was also documented as an unexpected adverse event, and the determination of whether these events were associated with the RIC device was adjudicated by research physicians. Serious adverse events (SAEs) in this study included death, life-threatening conditions, inpatient hospitalization or prolongation of existing hospitalization requiring medical/surgical intervention to prevent permanent impairment or damage, and other serious medical events.

### Statistical Analysis

The null hypothesis was that the recurrence rate of ischemic stroke/TIA within 3 months would be greater than the largest regression probability of recurrence (*P*_0_ = 12%). The alternative hypothesis was that the recurrence rate of ischemic stroke/TIA within 3 months would be less than the smallest regression probability of recurrence (*P*_*A*_= 6%) (14). To test our hypothesis, with an assumption of a type 1 error of 5%, a power of 80%, and an attrition rate of 10%, the sample size was estimated to be 165 (15).

Baseline categorical variables were listed as numbers (percentage), baseline normally distributed continuous variables were reported as means (standard deviation, SD), and non-normally distributed continuous variables were reported as medians (interquartile range, IQR).

We used multivariate logistic regression models to assess the association between the odds ratio (OR) of recurrent ischemic stroke/TIA occurring within 3 months with a compliance rate of RIC (≥50 vs. <50%), adjusting the antiplatelet strategy (dual vs. single antiplatelet), age (≥60 vs. <60 years), and sex (male vs. female) parameters. Moreover, we used repeated-measures analysis with mixed models to analyze NIHSS or handgrip strength changes associated with the RIC compliance rate (≥50 vs. <50%), antiplatelet strategy (dual vs. single antiplatelet), and follow-up visits (baseline, 1 month, and 3 months). To follow the parsimonious principle in variable selection for regression models, we only included the most crucial covariates that may affect recurrence or neurological behavior results. A *P*-value of < 0.05 was considered as statistically significant, and all statistical analyses were performed using SAS software, version 9.4 (SAS Institute, Carry, NC, USA).

## Results

### Study Patients

**Figure 1:**
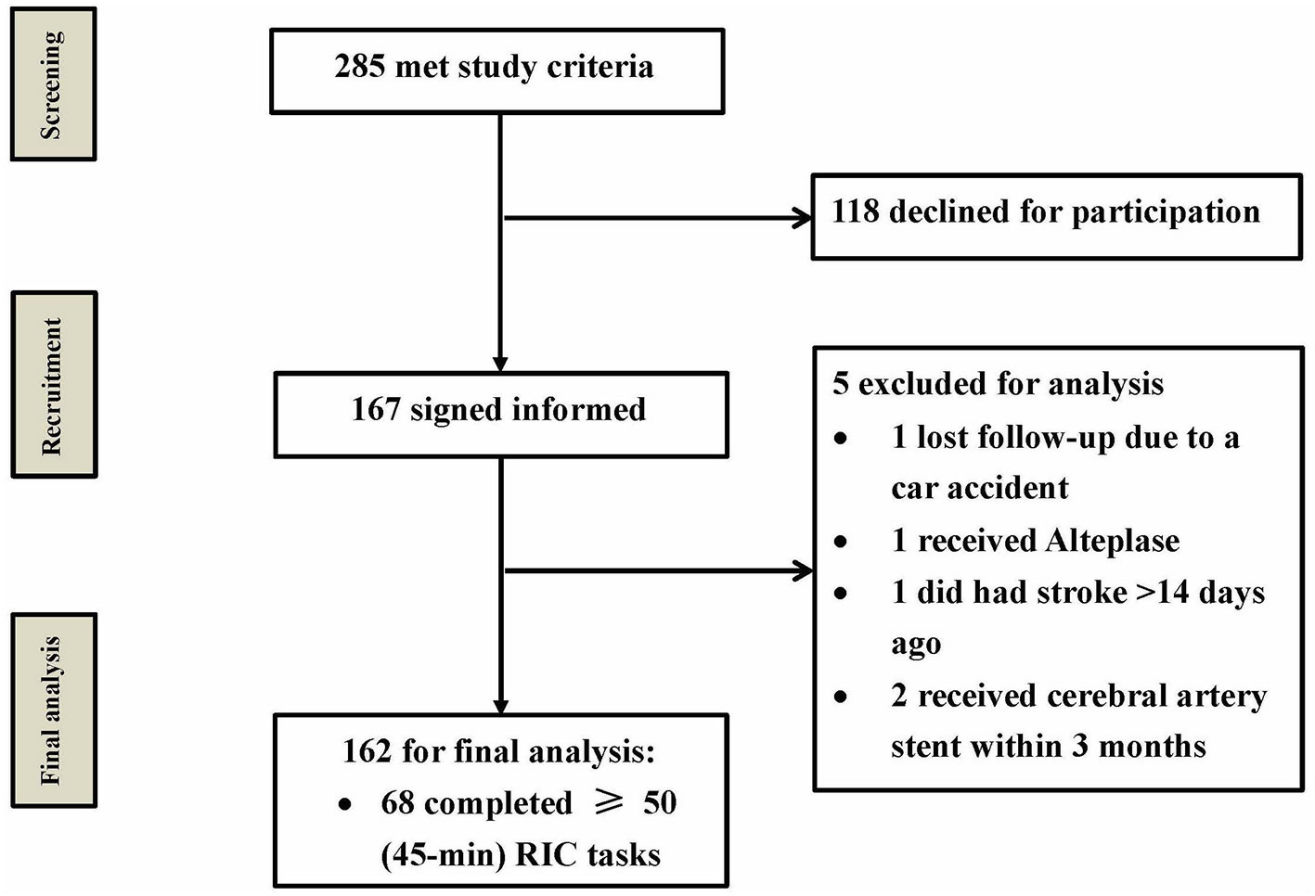
Study patient flow chart. From December 2016 to August 2017, a total of 285 patients met the study criteria, and 167 of them provided informed consent. Only 162 patients were included for final analysis, and five patients were excluded. RIC, remote ischemic conditioning.

From December 2016 to August 2017, 285 patients met the study criteria at the four enrollment centers, and 167 of them provided signed informed consent and were enrolled in the study. Ultimately, a total of 162 patients were included in the final analysis, whereas 118 patients declined to participate for various reasons, including skepticism of the study device or clinical trials, inability to commit to follow-up visits, and other non-specific reasons. Notably, five patients were excluded for the following reasons: one patient lost to follow-up due to a car accident; one patient received Alteplase meeting the exclusion criteria; one patient did not meet the inclusion criteria (had stroke >14 days ago); and two patients received cerebral artery stent within 3 months meeting the exclusion criteria (Figure 1).

The median age of the included patients was 58 years, and 74.7% (121/162) of them were male. 106 (65.4%) patients received dual antiplatelet therapy with the combination of aspirin and clopidogrel, 45 (27.8%) patients received aspirin and 11 (6.8%) received clopidogrel. The index event was AMIS in 153 patients (94.4%). The median NIHSS score was 1. ABCD^2^ score was 5 in the patients with TIA. Regarding medical history, 69.8% (113/162) of the included patients had hypertension, 36.4% (59/162) had diabetes, and 51.2% (83/162) were current or previous smokers. Furthermore, the median time from the onset of the qualifying event to RIC intervention was 7 days. Baseline blood biochemical test results of the included patients are listed in Table 1.

**Table 1:** Baseline clinicodemographic characteristics.

**Variables**	**All patients (***n*** = 162)**	**Dual antiplatelet (***n*** = 106)**	**Single antiplatelet (***n*** = 56)**
Age, median (IQR), years	58 (51–67)	59 (51–67)	58 (52–67)
Male, *n* (%)	121 (74.7)	83 (78.3)	38 (67.9)
Body mass index, median (IQR)	25.0 (23.0–27.7)	25.6 (23.4–27.7)	24.1 (22.3–26.6)
**Blood pressure, median (IQR), mmHg**			
Systolic	148 (135–162)	150 (135–162)	146 (130–164)
Diastolic	83 (75–94)	85 (80–94)	80 (73–93)
NIHSS, median (IQR)	1 (1–2)	1 (0–2)	2 (1–2)
Hand grip strength, median (IQR), kg^†^	22.5 (13.0–29.0)	23.6 (12.9–30.2)	20.0 (13.0–26.9)
**Medical history**, ***n*** **(%)**			
Hypertension	113 (69.8)	81 (76.4)	32 (57.1)
Hyperlipidemia	81 (50.0)	56 (52.8)	25 (44.6)
Diabetes	59 (36.4)	38 (35.9)	21 (37.5)
Ischemic stroke/TIA	37 (22.8)	20 (18.9)	17 (30.4)
Coronary heart disease	21 (13.0)	17 (16.0)	4 (7.1)
Intracranial hemorrhage	2 (1.2)	1 (0.9)	1 (1.8)
Atrial fibrillation/flutter	0	0	0
Current or previous smoking, *n* (%)	83 (51.2)	47 (44.3)	36 (64.3)
Family history of stroke, *n* (%)	60 (37.0)	45 (42.5)	15 (26.8)
Time to receive intervention, median (IQR), d	7 (5–9)	7 (5–9)	7 (5–10)
**Qualifying event**			
Acute minor ischemic stroke, *n* (%)	153 (94.4)	99 (93.4)	54 (96.4)
**TOAST classification**, ***n*** **(%)**			
Large-artery atherosclerosis	100 (65.4)	64 (64.7)	36 (66.7)
Cardioembolism	0 (0)	0 (0)	0 (0)
Small-vessel occlusion	50 (32.7)	33 (33.3)	17 (31.5)
Stroke of other determined etiology	0 (0)	0 (0)	0 (0)
Stroke of undetermined etiology	3 (2.0)	2 (2.0)	1 (1.9)
TIA, *n* (%)	9 (5.6)	7 (6.6)	2 (3.6)
ABCD^2^ (1) score, median (IQR)	5 (4–6)	5 (4–6)	5 (4–6)
**Blood Test**			
TChol, median (IQR), mmol/L	4.25 (3.70–4.90)	4.30 (3.78–184)	4.20 (3.55–3.92)
HDL, median (IQR), mmol/L	1.04 (0.88–1.30)	1.06 (0.88–1.30)	1.01 (0.85–1.27)
LDL, median (IQR), mmol/L	2.67 (2.07–3.16)	2.75 (2.18–3.19)	2.59 (2.07–3.13)
GLU, median (IQR), mmol/L	5.7 (4.7–7.7)	5.7 (4.9–7.7)	5.5 (4.7–8.0)
HbA_1C_, median (IQR), %^‡^	7.7 (6.7–9.1)	7.7 (6.7–9.1)	7.7 (6.7–9.1)
HCY, median (IQR), μmol/L	11.6 (10.0–15.9)	11.4 (9.9–16.5)	11.9 (9.8–15.2)

*IQR, interquartile range; NIHSS, NIH Stroke Scale; Tchol, total cholesterol; HDL, high-density lipoprotein cholesterol; LDL, low-density lipoprotein cholesterol; GLU, glucose; Hb A1C, hemoglobin A1C; HCY, homocysteine*.

† *Hand grip strength for patients with upper limb weakness without recurrent vascular events*.

𠈁 *HbA_1C_ test for patients with diabetes mellitus*.

### Primary Outcomes

Ischemic stroke/TIA occurred in six patients (3.7%) within 3 months. It rejects the null hypothesis and supports the alternative hypothesis. (i.e. the recurrence rate of ischemic stroke/TIA within 3 months is less than the smallest regression probability of recurrence of 6%). Among the six patients, three patients received dual antiplatelet therapy (Table 2), two patients received aspirin, and one patient received clopidogrel alone. Regarding compliance to RIC, Three patients are being compliant while the other 3 patients are not compliant.

**Table 2:** Study outcomes.

**Outcomes**	**All patients (***n*** = 162)**	**Antiplatelet group**		**Compliance to RIC**	
		**Dual antiplatelet** **(***n*** = 106)**	**Single antiplatelet** **(***n*** = 56)**	**≥ 50% compliance** **(***n*** = 68)**	** <50% compliance** **(***n*** = 94)**
**Primary efficacy outcome**					
Ischemic stroke/TIA within 3 months, *n* (%)	6 (3.7)	3 (2.8)	3 (5.4)	3 (4.4)	3 (3.2)
**Secondary efficacy outcomes**					
Ischemic stroke/TIA within 1 month, *n* (%)	2 (1.2)	1 (0.9)	1 (1.8)	0	2 (2.1)
Stroke, myocardial infarct or death from cardiovascular causes, *n* (%)	6 (3.7)	3 (2.8)	3 (5.4)	3 (4.4)	3 (3.2)
Hemorrhagic stroke, *n* (%)	0 (0)	0 (0)	0 (0)	0 (0)	0 (0)
Estimated NIHSS change from baseline to 1 month, Mean (SD)^†^	−0.8 (0.1)	−0.6 (0.1)	−0.9 (0.2)	−0.7 (0.1)	−0.7 (0.1)
Estimated NIHSS change from baseline to 3 months, mean (SD)^†^	−1.0 (0.1)	−1.0 (0.1)	−1.1 (0.1)	−1.0 (0.1)	−1.0 (0.1)
mRS ≤ 1 at 1 month, *n* (%)^†^	108 (75.0)	74 (78.7)	34 (68.0)	51 (75.3)	57 (79.8)
mRS ≤ 1 at 3 months, *n* (%)^†^	107 (74.8)	73 (78.5)	34 (68.0)	50 (74.6)	57 (79.8)
mRS at 1 month, median (IQR)^†^	1 (0–2)	1 (0–1)	0 (0–2)	1 (0–1)	0 (0–1)
mRS at 3 months, median (IQR)^†^	1 (0–2)	1 (0–1)	0 (0–2)	1 (0–1)	0 (0–1)
BI ≥ 95 at 1 month, *n* (%)^†^	133 (89.3)	89 (89.9)	44 (88.0)	65 (89.9)	69 (88.2)
BI ≥ 95 at 3 months, *n* (%)^†^	132 (97.8)	88 (98.9)	44 (95.7)	64 (98.7)	69 (97.1)
BI at 1 month, median (IQR)^†^	100 (100–100)	100 (100–100)	100 (100–100)	100 (100–100)	100 (100–100)
BI at 3 months, median (IQR)^†^	100 (100–100)	100 (100–100)	100 (100–100)	100 (100–100)	100 (100–100)
Estimated hand grip strength change from baseline to 1 month, mean (SD), Kg^†^	4.3 (0.8)	4.3 (0.9)	4.2 (1.3)	3.4 (1.1)	3.5 (1.1)
Estimated hand grip strength change from baseline to 3 months, mean (SD), Kg^†^	4.6 (0.8)	4.5 (0.9)	4.7 (1.4)	4.7 (1.5)	4.8 (1.5)
**Safety outcomes**, ***n*** **(%)**					
Pain (upper limb)	44 (27.2)	27 (25.5)	17 (30.4)	25 (36.8)	19 (20.2)
Petechia (upper limb)	26 (16.0)	20 (18.9)	6 (10.7)	8 (11.8)	18 (19.1)
Heart palpitation	5 (3.1)	4 (3.8)	1 (1.8)	1 (1.5)	4 (4.3)
Superficial venous thrombosis (upper limb)	1 (0.6)	1 (0.9)	0 (0)	0 (0)	1 (1.0)
Hand cramps	1 (0.6)	0 (0)	1 (1.8)	1 (14.7)	0 (0)
Any bleeding	0 (0)	0 (0)	0 (0)	0 (0)	0 (0)

*RIC, remote ischemic conditioning; IQR, interquartile range; SD, standard deviation; NIHSS, NIH Stroke Scale; mRS, modified Rankin Scale; BI, Barthel Index; RIC, remote ischemic conditioning*.

† *Tests among patients who did not have a recurrence event and visited the outpatient department in person*.

### Secondary Efficacy Outcomes

Ischemic stroke/TIA occurred in one patient (0.9%) under dual antiplatelet therapy, and one patient (1.8%) under single antiplatelet therapy (aspirin, to be specific) within 1 month. None of the included patients had any other vascular events or hemorrhagic stroke. We collected neurological outcomes data from 143 patients who visited the outpatient department in person, showing that 74.8% (107/143) of patients had a favorable outcome (mRS score of 0 or 1) at 3 months, with a median mRS score of 1 (0–2) and Barthel index of 100 (100–100). The mean change in NIHSS score in the study patients was −0.8 and −1.0 within 1 and 3 months, respectively. The mean change in grip strength at the affected upper limbs was 4.3 and 4.6 Kg within 1 and 3 months, respectively (Table 2).

### Safety Outcomes

Upper limb pain was reported to be the most common (44/162, 27.2%) local adverse event, followed by upper limb petechia (26/162, 16.0%), heart palpitation (5/162, 3.1%), and superficial venous thrombosis within the upper limb (1/162, 0.6%). No bleeding events occurred. No SAEs were observed.

### Compliance

Only 68 patients (42.0%) completed ≥ 50% of the long-term, regular, 45-min RIC sessions using real-time 4G signal data (Table 2). They were 43 men (63%) and the median age of the 68 patients was 61 years. Three (4.4%) patients had the primary outcomes within three months, and none had recurrence within 1 month (Table 2).

### Subgroup Analysis

**Figure 2:**
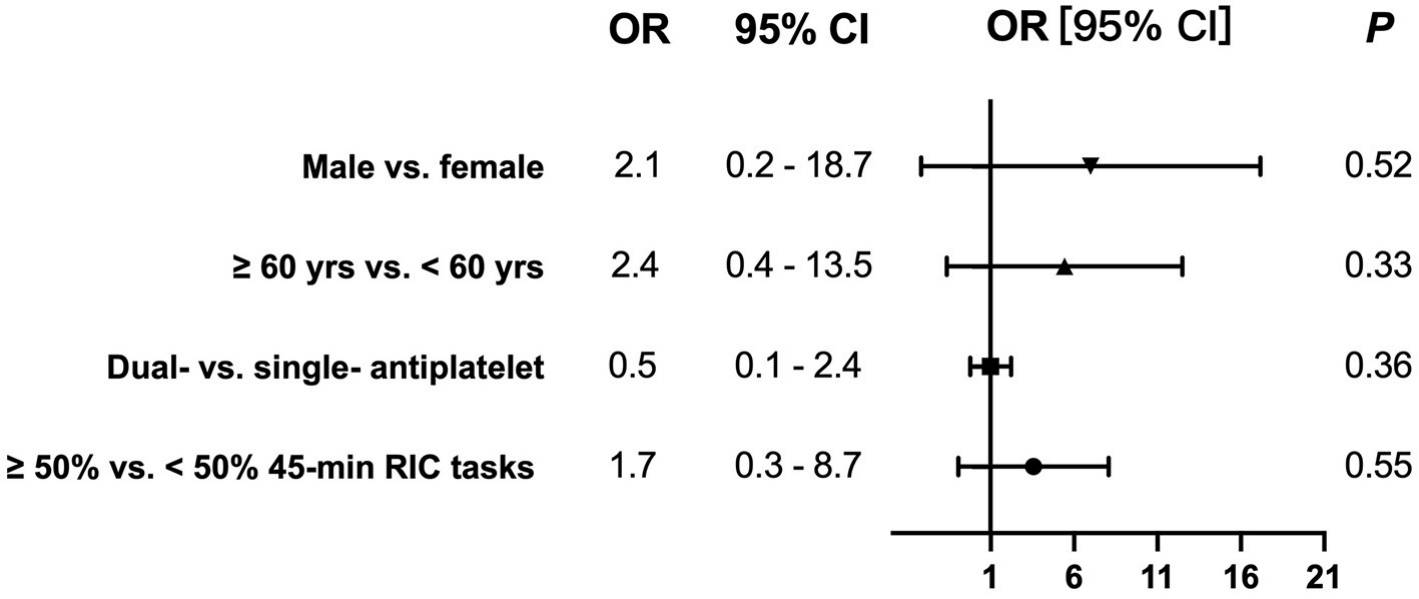
No significant odds ratios (ORs) of recurrent ischemic stroke/TIA within 3 months were detected on comparison of the subgroups completing ≥50% the 45-min RIC tasks and those who did not [OR: 1.7, 95% confidence interval (CI): 0.3–8.7, *P* = 0.55], after adjusting for age (≥60 vs. <60 years), sex (male vs. female), and antiplatelet strategy (dual vs. single antiplatelet).

No significant difference of recurrent ischemic stroke/TIA within 3 months were detected on comparison of the subgroups completing ≥50% of the 45-min RIC tasks and those who did not (OR: 1.7, 95% CI: 0.3–8.7, *P* = 0.55), after adjusting for age (≥60 vs. <60 years), sex (male vs. female), and antiplatelet strategy (dual vs. single antiplatelet) (Figure 2). In the multiple-variable model, age (≥60 vs. <60 years), sex (male vs. female) or antiplatelet strategy (dual vs. single antiplatelet) was not associated with the risks of having a recurrence.

In the repeated-measures models consisting variables of antiplatelet strategy (dual vs. single antiplatelet), the compliance rate of 45-min RIC (≥50 vs. <50%), and follow-up visits (baseline, 1 month and 3 months), NIHSS at 3 months was significantly improved, as compared to baseline (−1.0 ± 0.1, 95% CI: −1.2 to −0.9; *P* < 0. 001), although antiplatelet strategy and compliance rate of 45-min RIC were not associated with NIHSS change in this model (*P* = 0.60 and 0.76, respectively). Similarly, handgrip strength was estimated to increase by 4.6 ± 0.8 kg from baseline to 3 months in patients with unilateral upper limb weakness resulting from the index event without any vascular events (95% CI 3.0–6.0; *P* < 0. 001) (Table 2); however, antiplatelet strategy and compliance rate of 45-min RIC were not associated with the handgrip strength change in this model (*P* = 0.24 and 0.98, respectively).

## Discussion

The PICNIC-One study found that regular 3-month twice daily RIC could significantly prevent recurrent ischemic stroke or TIA in patients with AMIS or moderate-to-high risk TIA, showing a recurrence rate of 3.7%. However, the compliance to RIC was not great as only 42.0% of the included patients completed 50% of the study procedures (i.e., 180 sessions of the 45-min RIC), RIC was demonstrated to be safe among the patients due to a low incidence of adverse events.

Only two-thirds of patients with AMIS/TIA were prescribed with dual antiplatelet therapy, despite the fact that the Chinese secondary prevention of stroke guidelines have been in place for 3 years (5). This proved the need to conduct this single-arm study prior to a randomized controlled Phase II study to elucidate the real-world practices of the recommended antiplatelet strategies. In this study, the recurrence rate of ischemic stroke/TIA within 3 months was 3.7%, which appears to be lower than that in the CHANCE study (3). It suggests there is likely a signal using RIC as an adjunctive therapy on top of short-duration use of dual-antiplatelets, but we have to interpret the data with great caution. However, this comparison should be interpreted with caution because the study population in the PICNIC-One study could be different although PICNIC study adopted the inclusion criteria from the CHANCE study. Although age, sex, or medical histories weresimilar between the two studies, only 5.6% of patients suffered TIA as compared to 27.9% patients having TIA in the CHANCE study. The antiplatelet strategy can interfere with the outcomes, and its usage are different between the two studies. For example, aspirin alone was put on 27.8% of patients, clopidogrel alone was prescribed to 6.8% of patients and dual-antiplatelets accounted for 65.4% of patients in the PICNIC-One study. In the CHANCE study, half of the patients were randomized into dual-antiplatelet strategy and the other half were assigned to aspirin alone.

Contrary to our expectations, the compliance of RIC was not good, with fewer than half of the patients completing half of the RIC sessions. It raised a tolerability issue as 27.2% of patients reported pain as the most common side effect. Interestingly the group being more compliant are more likely to report pain as a side effect as compared with the less compliant group (36.8 vs. 20.2%). Notably, long-term RIC has been reported to be well-accepted, with a high compliance rate in 15 prior small clinical trials (10, 11, 16–28), three of which were carried out at the same center (10, 11, 26), however, we have to point out that these compliance rate is based on subjective self-report from patient vs. quantitative objective measures by this study In PICNIC-One study, four study sites actively recruited patients in multiple regions of China. The electric auto-control RIC device had the capacity to upload application data simultaneously through 4G signals, allowing the study team to calculate the compliance rate with objective data. With the objective application data from four centers, the PICNIC-One study advanced the current knowledge regarding the feasibility and compliance of applying long-term regular RIC sessions in this subset of stroke population patients.

Although prior clinical studies have indicated that long-term, regular RIC (twice a day for at least 90 consecutive days or 180 sessions) was effective in reducing stroke recurrence in the patients with symptomatic intracranial artery stenosis (10, 11). There is no statistical difference in primary efficacy outcomes between the more compliant and less compliant group in PICNIC-one study although the study is not powered to demonstrate such difference. We do not know whether less than three months of adjunctive RIC therapy (or <180 sessions) are adequate to prevent stroke recurrence. It remains unknown about the optimal dose of RIC in secondary stroke prevention. As compliance and tolerability is critical issue in this study, it is important to design a dosing-selection study as the next step to better understand the dose, preliminary efficacy and to improve the compliance rate.

In addition to its potential efficacy, the PICNIC-One study also provided evidence of safety profile associated with long-term RIC, reporting no incidences of SAEs. To the best of our knowledge, this is the first study to report RIC-related heart palpitation (3.1%), hand cramps (0.6%), and superficial venous thrombosis (0.6%). We did not find any relationship between adverse events of RIC and functional recovery of patients, which should be tested in the next step.

Despite the important findings of this study, certain limitations were noted. First, while this was a single-arm, not sham-controlled study. The purpose of this study was to get a first-hand experience on using RIC in this stroke subpopulation and to collect preliminary data to better calculate the sample size for the next study. Single-arm study has the advantage of completing the study procedure faster and more cost-effective than a sham-controlled study. Second, the compliance rate needs to be improved. In our next study, we plan to conduct regular daily RIC at home with the support of mobile health in order to improve the patient compliance rate, as described in a previous study (29). Lastly, the present study is not designed to demonstrate the associations between RIC compliance and efficacy outcomes. The optimal dose of RIC remains unknown.

In conclusion, RIC is a safe add-on procedure with a potential benefit in reducing cerebrovascular events in a subgroup of patients with non-cardiogenic AMIS/TIA; however, its compliance is not high and needs to be improved in the future study. Our study provided critical preliminary data to plan a large sample size, randomized controlled clinical study to systematically investigate the safety and efficacy of RIC in this unique stroke patient population.

## Data Availability Statement

The original contributions presented in the study are included in the article/supplementary material, further inquiries can be directed to the corresponding author/s.

## Ethics Statement

The studies involving human participants were reviewed and approved by Xuanwu Hospital. The patients/participants provided their written informed consent to participate in this study.

## Author Contributions

SLiu: study design and conduct and manuscript preparation. ZG, RM, and HS: study design and conduct. TT, RC, YS, QF, FJ, QZ, JDi, XiaH, QM, KD, SX, ZY, JDu, CC, XC, XinH, and SLi: study conduct. BO and WZ: study design. XJ: study design and conduct and manuscript revise. WF: study design and manuscript revise. All authors contributed to the article and approved the submitted version.

## Funding

This study was funded by the Chang Jiang Scholars Program (Grant No. T2014251), the National Key R&D Program of China (Grant No. 2017YFC1308401), the Beijing Municipal Administration of Hospitals Clinical Medicine Development of Special Funding Support (Grant No. ZYLX201706), the Beijing Municipal Administration of Hospitals' Mission Plan (Grant No. SML20150802), Ten Thousand Talent Program, Beijing Municipal Administration of Hospitals' Mission Plan (Grant No. SML20150802), Talent Introduction Project of the Beijing Bureau of Foreign Expert (Grant No. BJ2018001), and the National Natural Science Foundation of China (Grant No. 82001242).

## Conflict of Interest

The authors declare that the research was conducted in the absence of any commercial or financial relationships that could be construed as a potential conflict of interest.

## Publisher's Note

All claims expressed in this article are solely those of the authors and do not necessarily represent those of their affiliated organizations, or those of the publisher, the editors and the reviewers. Any product that may be evaluated in this article, or claim that may be made by its manufacturer, is not guaranteed or endorsed by the publisher.
